# Opioid-free strategies for patient-controlled intravenous postoperative analgesia: a review of recent studies

**DOI:** 10.3389/fphar.2024.1454112

**Published:** 2024-10-31

**Authors:** Xin Luo, Pan-Guo Rao, Xing-Heng Lei, Wen-Wen Yang, Bao-Zhen Liao, Rui Guo

**Affiliations:** ^1^ Gannan Medical University, Ganzhou, Jiangxi, China; ^2^ Department of Anesthesiology, First Affiliated Hospital of Gannan Medical University, Ganzhou, Jiangxi, China

**Keywords:** postoperative pain, opioids, non-steroidal anti-inflammatory drugs, opioid-free, postoperative patient-controlled intravenous analgesia

## Abstract

Postoperative pain management has consistently been a critical topic in the medical field, with patient-controlled intravenous analgesia (PCIA) being one of the most commonly utilized methods for postoperative analgesia. Currently, opioids remain the primary choice for PCIA in clinical practice. However, in recent years, an increasing number of studies have explored analgesic strategies aimed at reducing or eliminating the use of opioids in PCIA to mitigate the associated side effects and dependence. This article systematically reviews the progress of research on opioid-free analgesic strategies in PCIA through a comprehensive analysis of relevant literature.

## 1 Introduction

Postoperative pain is one of the major challenges patients often encounter. It not only affects patients’ quality of life and recovery speed but also can lead to a series of complications, prolonging hospital stays. To effectively manage postoperative pain, various analgesic methods and strategies have been extensively researched and applied. Among these, PCIA is a popular and proven effective method, favored by both medical professionals and patients. Traditionally, opioids have been considered the most effective drugs in PCIA. However, their use is associated with multiple risks, including respiratory depression, nausea and vomiting, as well as potential for dependency and abuse. Given these issues, the search for safer and more effective alternatives has become a focal point in current research on postoperative analgesia. In recent years, non-opioid drugs and their combinations have demonstrated potential in PCIA and are considered effective strategies for reducing or eliminating opioid use. This paper aims to systematically summarize the research progress on combination strategies that replace opioids in postoperative PCIA, evaluating the effectiveness, advantages, and limitations of various approaches, and exploring future research directions. The goal is to provide new insights and strategies for postoperative pain management.

## 2 Methods

### 2.1 Literature search and screening process

The literature search was conducted by the primary authors Xing-Heng Lei and Wen-Wen Yang, who utilized databases such as PubMed, Cochrane Library, Web of Science, and Google Scholar. They conducted searches and screenings using a variety of keywords, including but not limited to “opioid-free analgesia,” “opioid drugs,” “patient-controlled intravenous analgesia,” “non-steroidal anti-inflammatory drugs,” “intravenous analgesia,” “postoperative pain,” and “acute pain.” Additionally, relevant citations were traced to collect recent research literature on opioid-free patient-controlled intravenous analgesia in postoperative settings, providing a comprehensive summary. The search timeframe spanned from January 2010 to September 2024. A preliminary screening yielded 490 articles, which were subsequently evaluated based on titles, abstracts, and full texts, resulting in the inclusion of 71 studies that met the established criteria.

### 2.2 Literature review

The selected articles were independently reviewed by two reviewers, Bao-Zhen Liao and Pan-Guo Rao. They performed a quality assessment of each article to confirm adherence to the inclusion criteria, which encompassed the characteristics of study participants and the validity of study design.

### 2.3 Discrepancy resolution

**FIGURE 1:**
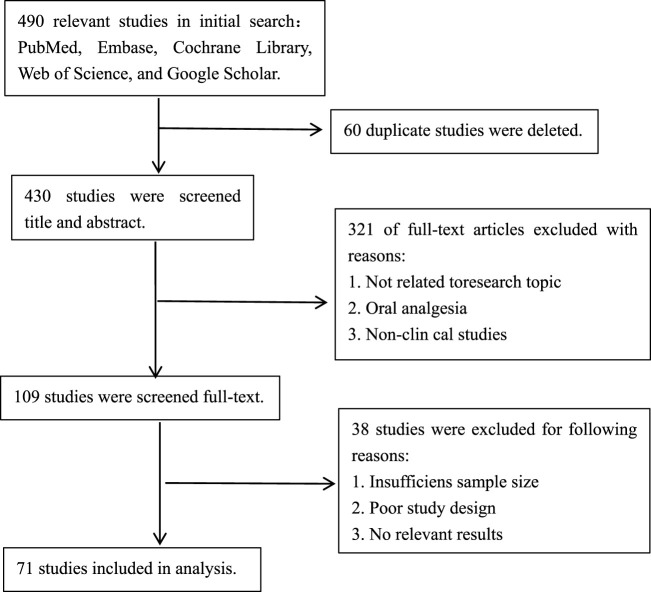
Literature search and screening process.

In the event of any discrepancies during the review process, the research team convened a meeting to reach a final decision, led by researchers Xin Luo and Rui Guo. All disputed articles and the resulting decisions were documented to ensure transparency and fairness. The flowchart of literature search and screening is shown in Figure 1.

## 3 Application of opioids in PCIA

### 3.1 Introduction to opioids

Opioid analgesics are the primary drugs used in PCA therapy due to their excellent analgesic effects and rapid onset of action, making them the most widely used analgesics during the perioperative period. As the most common central analgesics, opioids play a crucial role in both acute and chronic pain management due to their potent analgesic properties.

Opioids can be classified into three categories based on their source and manufacturing process: natural alkaloids, semi-synthetic drugs, and synthetic drugs. In clinical practice, the primary natural opioid utilized is morphine. Additionally, synthetic opioids are commonly employed, including meperidine and the fentanyl family (fentanyl, alfentanil, remifentanil, and sufentanil). The analgesic effects of opioids are primarily achieved through their binding to specific receptors in the central nervous system and other tissues. These opioid receptors are mainly categorized into four types: μ, δ, κ, and σ, which are coupled with G proteins. Each receptor type has different subtypes and acts on the nervous system through various mechanisms.

Opioids exert their effects by inhibiting voltage-gated calcium channels and activating inwardly rectifying potassium channels. Upon binding to their receptors, they selectively inhibit the transmission of certain excitatory neural impulses, thereby alleviating the body’s perception of pain and the accompanying psychological and behavioral responses (Wang et al., 2023).

### 3.2 Side effects of opioid analgesia

The clinical use of opioids has evolved from the initial use of morphine to pethidine and later to the fentanyl family, with continuous improvements in the drugs. However, related adverse reactions and complications still persist. These include respiratory depression, gastrointestinal reactions, urinary retention, skin itching and rashes, and in severe cases, can lead to patient death. In clinical practice, opioids also present issues such as tolerance, addiction, withdrawal symptoms, and hyperalgesia (El-Kefraoui et al., 2020). The side effects of opioids in PCIA are summarized in Table 1.

**TABLE 1:** Side effects of opioids in PCIA.

Side effects of opioids	References
Respiratory depression	Baldo (2021); Jehangir et al. (2020); Baertsch et al. (2021)
Gastrointestinal side effects (nausea and vomiting, bloating and constipation)	Smith and Laufer (2014); Webster (2015)
Urinary retention	Tischler et al. (2016); de Boer et al. (2017)
Skin itching and rash	Golembiewski (2013); Smith et al. (2023); Wang et al. (2021)
Tolerance and addiction	Volkow et al. (2019)
Withdrawal symptoms	Kosten and Baxter (2019)
Hyperalgesia	Mercadante et al. (2019)
Sleep disturbances	Finlay et al. (2023)
Immunosuppression	Sun et al. (2023); Fan et al. (2017)

## 4 Common non-opioid intravenous analgesics

Several non-opioid intravenous analgesics are widely used in clinical practice, including non-steroidal anti-inflammatory drugs (NSAIDs), α2-adrenergic receptor agonists, N-methyl-D-aspartate (NMDA) receptor antagonists, gabapentinoids, sodium channel blockers, and corticosteroids. Each drug class has distinct mechanisms of action, and rational combinations can provide effective multimodal analgesia for postoperative patients.

### 4.1 Non-steroidal anti-inflammatory drugs (NSAIDs)

NSAIDs are frequently used to manage postoperative pain by inhibiting cyclooxygenase (COX) enzymes, thereby reducing the synthesis of prostaglandins, which mediate inflammation and pain. Common NSAIDs in anesthesia include flurbiprofen axetil, parecoxib sodium, and ketorolac tromethamine. Compared to other analgesics, NSAIDs provide both anti-inflammatory and analgesic effects, making them first-line agents for mild to moderate pain. However, their use is associated with gastrointestinal, renal, and cardiovascular side effects, particularly an increased risk of gastrointestinal bleeding and potential surgical site bleeding (Bongiovanni et al., 2021).

#### 4.1.1 Flurbiprofen axetil

Flurbiprofen axetil, a non-selective NSAID, inhibits both COX-1 and COX-2. It is delivered via a lipid microsphere carrier that enhances its targeting to inflamed tissues. Flurbiprofen axetil offers several advantages, including targeted drug delivery, controlled release, and avoidance of gastrointestinal complications (Zhang et al., 2018). Compared to oral NSAIDs, intravenous flurbiprofen axetil provides rapid onset and prolonged duration of action without affecting the central nervous system or respiratory function (Hersh et al., 2020). These characteristics make it suitable for controlling acute postoperative pain, particularly in patients at risk of gastrointestinal complications (Geng et al., 2015).

#### 4.1.2 Parecoxib sodium

Parecoxib sodium is a selective COX-2 inhibitor that is rapidly converted to its active form, valdecoxib, following administration. Its COX-2 selectivity reduces the risk of gastrointestinal irritation and bleeding while providing effective analgesia and anti-inflammatory effects. Compared to non-selective NSAIDs, parecoxib sodium has the advantage of avoiding platelet dysfunction, making it particularly beneficial for patients who are at risk of postoperative bleeding (Gehling et al., 2010).

#### 4.1.3 Ketorolac tromethamine

Ketorolac tromethamine is a potent non-selective COX inhibitor that provides significant analgesic effects, especially for moderate to severe postoperative pain. Its strong analgesic properties make it a suitable alternative to opioids, particularly in patients requiring rapid pain relief (Wu et al., 2022). However, ketorolac’s use is limited by its higher risk of gastrointestinal side effects and potential for increased bleeding, necessitating careful monitoring in the postoperative setting (Ying et al., 2022).

### 4.2 α2-Adrenergic receptor agonists

α2-adrenergic receptor agonists, such as dexmedetomidine, provide analgesic, sedative, and anxiolytic effects through the inhibition of norepinephrine release in the central nervous system (Peng et al., 2017). Dexmedetomidine’s ability to reduce opioid and anesthetic requirements without causing respiratory depression makes it an attractive option for postoperative pain management (Lin et al., 2023). However, its side effects, including bradycardia and hypotension, require careful monitoring, especially in patients with cardiovascular risk factors. Compared to NSAIDs, dexmedetomidine is less likely to cause gastrointestinal complications but may not be suitable for all patients due to its hemodynamic effects (Tang et al., 2020).

### 4.3 NMDA receptor antagonists

NMDA receptor antagonists, such as ketamine, offer analgesic effects by preventing central sensitization and hyperalgesia. Although ketamine is effective in reducing postoperative pain and opioid consumption, its use is limited by side effects, including excessive salivation, nausea, and psychiatric symptoms such as hallucinations and delirium (Zhang A. et al., 2024). Esketamine, the S-enantiomer of ketamine, offers more potent analgesic effects and a better side-effect profile compared to ketamine, although psychiatric side effects still limit its broader use (Han et al., 2022). Both drugs are valuable adjuncts in opioid-sparing regimens, but their psychiatric effects necessitate careful patient selection.

### 4.4 Dextromethorphan

Dextromethorphan, a centrally acting NMDA receptor antagonist, has been shown to elevate the pain threshold without inducing respiratory depression. Although not a direct analgesic, it can reduce nociceptive sensitivity, particularly when administered prior to painful stimuli (Martin et al., 2019). Its opioid-sparing effect makes it a useful adjunct in postoperative analgesia, particularly for reducing the overall opioid requirement and associated side effects (Chia et al., 1999).

### 4.5 Magnesium sulfate

Magnesium sulfate acts as an NMDA receptor antagonist, reducing central sensitization. Adjunctive use of magnesium sulfate in postoperative analgesia has been shown to reduce opioid consumption and enhance pain control (Song et al., 2011). However, its use is limited by the need for real-time monitoring of magnesium levels due to risks such as bradycardia and hypermagnesemia. Further research is required to establish optimal dosing regimens for safe and effective use in postoperative settings (Sousa et al., 2016).

### 4.6 Gabapentinoids

Gabapentinoids, such as gabapentin and pregabalin, are calcium channel blockers that prevent hyperalgesia and central sensitization by inhibiting neurotransmitter release. Pregabalin is generally considered more effective and better tolerated than gabapentin, although both drugs have been associated with increased risk of respiratory depression when used in combination with opioids (Evoy et al., 2021; Huang et al., 2021). Gabapentinoids can improve sleep and reduce anxiety, making them valuable in multimodal analgesia for postoperative pain, particularly in patients with chronic pain conditions (Bockbrader et al., 2010). However, their use must be carefully tailored in patients with renal impairment due to their renal clearance.

### 4.7 Other adjuvant analgesics

Local anesthetics, such as lidocaine, provide membrane stabilization by inhibiting sodium channels, reducing nociceptive transmission, and alleviating hyperalgesia (Alebouyeh et al., 2014). Intravenous lidocaine has demonstrated efficacy in reducing postoperative opioid consumption and improving recovery outcomes, particularly in abdominal surgery (Kranke et al., 2015). Glucocorticoids, such as dexamethasone, are widely used for their anti-inflammatory and antiemetic effects (Chu et al., 2014). When combined with opioids, glucocorticoids have been shown to extend the duration of opioid analgesia and reduce opioid consumption, although long-term use poses risks such as immunosuppression (Lee et al., 2002; Ryoo et al., 2015). The commonly used non-opioid medications in PCIA are shown in Table 2.

**TABLE 2:** The commonly used non-opioid medications in PCIA.

Non-opioid analgesics	References
NSAIDs	
- Flurbiprofen axetil	Zhang et al. (2018); Hersh et al. (2020); Geng et al. (2015)
- Parecoxib sodium	Gehling et al. (2010)
- Ketorolac tromethamine	Wu et al. (2022); Ying et al. (2022)
α_2_-adrenergic receptor agonists	
- Dexmedetomidine	Peng et al. (2017); Lin et al. (2023); Tang et al. (2020)
NMDA receptor antagonists	
- Ketamine	Zhang A. et al. (2024)
- Esketamine	Han et al. (2022)
- Dextromethorphan	Martin et al. (2019); Chia et al. (1999)
- Magnesium sulfate	Song et al. (2011); Sousa et al. (2016)
Gabapentins	
- Gabapentin	Bockbrader et al. (2010)
- Pregabalin	Evoy et al. (2021); Huang et al. (2021)
Local anesthetics	
- Lidocaine	Alebouyeh et al. (2014); Kranke et al. (2015)
Glucocorticoids	
-Dexamethasone	Chu et al. (2014); Lee et al. (2002); Ryoo et al. (2015)

## 5 Current status and challenges of opioid-free PCIA in clinical practice

### 5.1 Current clinical application of non-opioid PCIA

The use of opioid-free medications in PCIA, while not as effective for pain relief as opioids, can adequately meet clinical analgesic needs for postoperative mild to moderate pain when appropriately selected and optimally combined. Common procedures associated with mild pain, such as inguinal hernia repair, varicose vein ligation, and laparoscopic surgeries, can be effectively managed with opioid-free PCIA. Moderate pain procedures like knee and below-knee surgeries, shoulder and back surgeries, hysterectomies, and maxillofacial surgeries also respond well to non-opioid PCIA. Postoperative pain following thyroid surgery, which mainly manifests as pain during swallowing and subsequent difficulty in eating, is classified as mild pain and can also be managed with opioid-free PCIA (Gerbershagen et al., 2013).

The combination of two non-opioid intravenous analgesics in PCIA, along with an optimized regimen, can effectively provide analgesia for these types of surgeries. Additionally, incorporating single-shot nerve blocks for certain surgical sites as part of a multimodal analgesia approach can achieve satisfactory pain control, demonstrating the feasibility of opioid-free PCIA.

However, the use of opioid-free PCIA for procedures associated with severe pain, such as thoracotomies, laparotomies, total knee or hip replacements, and major vascular surgeries, still faces limitations. The primary challenge is that opioid-free analgesics may not sufficiently manage the high-intensity postoperative pain, particularly during peak pain periods. Therefore, for such surgeries, combining non-opioid analgesics with opioids or other more potent analgesic strategies may be necessary to enhance pain control efficacy and patient comfort.

### 5.2 The medication regimen or combination for opioid-free PCIA

NSAIDs are the most widely used medications for postoperative analgesia aside from opioids. Therefore, in opioid-free PCIA, a common combination involves NSAIDs paired with another non-opioid medication (Pergolizzi et al., 2021). For instance, NSAIDs combined with dexmedetomidine have been used in PCIA. A study on postoperative pain following thyroid surgery (Ma et al., 2017) showed that the combination of dexmedetomidine and flurbiprofen axetil significantly improved pain control after general anesthesia for thyroid surgery, reduced agitation and postoperative cognitive dysfunction, enhanced immune function, and promoted surgical wound healing.

Additionally, a study on postoperative analgesia following joint replacement surgery (Ohnuma et al., 2020) found that the combination of NSAIDs and gabapentin for PCIA resulted in pain scores comparable to those observed with opioid monotherapy. This combination also reduced the use of opioids and lowered the incidence of postoperative pulmonary complications. Research indicates that the combination of NSAIDs with ketamine can synergistically increase patients’ pain thresholds, reduce pain perception, decrease inflammation, and alleviate clinical symptoms of hyperalgesia (Mitra et al., 2018). NSAIDs can also counteract tramadol’s constipating effects, and their combined use can even boost macrophage spontaneous release of pro-inflammatory cytokines, enhancing cellular immune function (Filipczak-Bryniarska et al., 2023). The medication regimen or combination for opioid-free PCIA is presented in Table 3.

**TABLE 3:** The medication regimen for opioid-free PCIA.

Drug(s)	Surgery	References
NSAIDs (Ketorolac, acetaminophen, flurbiprofen)	radical cystectomy, partial mastectomies	Yu et al. (2018); Nonaka et al. (2016)
NSAIDs combined with dexmedetomidine	laparoscopic cholecystectomy, radical operation of lung cancer	Du et al. (2019); Zong et al. (2021)
NSAIDs combined with gabapentin	joint replacement surgery, sleeve gastrectomy surgical, total knee arthroplasty	Ohnuma et al. (2020); Khan et al. (2019); Eckhard et al. (2019)
NSAIDs combined with ketamine	Spinal fusion surgery	Mitra et al. (2018)
Ketamine or S-ketamine	Cesarean section operation, elective VATS lobectomy, hip fracture surgery	Han et al. (2022); Qi et al. (2024); Cai et al. (2024)

### 5.3 Advantages of opioid-free analgesia via PCIA in postoperative pain management

Research suggests that the use of entirely non-opioid analgesics via PCIA is feasible for managing postoperative pain in specific cases (Baboli et al., 2020). This approach effectively controls surgical-induced inflammatory responses and reduces occurrences of respiratory depression, nausea, vomiting, and pain hypersensitivity. Moreover, it diminishes opioid dependence in patients typically prescribed opioids post-discharge, enhances postoperative recovery quality, shortens hospital stays, reduces medical costs, and conserves healthcare resources (Savoia and Scibelli, 2022). Additionally, it may potentially mitigate postoperative tumor recurrence and metastasis (Forget et al., 2010). Combinations of non-opioid analgesics, such as nonsteroidal anti-inflammatory drugs (NSAIDs), NMDA receptor antagonists, and dexmedetomidine, offer superior intraoperative analgesia without the use of opioids, with minimal associated side effects (Zhang S. et al., 2024). Especially for elderly, frail, or critically ill patients, opioid-free PCIA has less impact on consciousness and respiration, providing greater safety compared to opioid-based PCIA.

### 5.4 Limitations of opioid-free PCIA

Opioid-free PCIA has its limitations. Unlike opioid drugs, non-opioid medications have a narrower therapeutic window and are subject to the ceiling effect. The most notable limitation is that their analgesic efficacy is generally inferior to opioids, particularly for severe pain. However, with the advancement of multimodal analgesia and nerve block techniques, opioid-free PCIA combined with effective nerve blocks can meet postoperative pain management needs for certain surgeries involving severe pain.

Additionally, the potential adverse effects and interactions of non-opioid drugs cannot be overlooked. For instance, dexmedetomidine can cause bradycardia and hypotension (Xie K. et al., 2023; Dong et al., 2017), NSAIDs may lead to gastrointestinal bleeding and renal dysfunction (Xie H. et al., 2023; Xu et al., 2023), and ketamine can induce hallucinations and agitation (Guo et al., 2023; Zhang et al., 2023a; Zhang et al., 2023b). It is also essential to be mindful of other potential adverse effects of these medications. Although studies indicate that commonly used drugs such as NSAIDs, dexmedetomidine, ketamine, magnesium sulfate, and lidocaine do not have compatibility contraindications, there is a lack of research on their *in vivo* interactions. Previous studies have found that the incidence of adverse interactions increases exponentially with the number of anesthetic drugs used (Fiore et al., 2022). Therefore, further research is needed to clarify the safety of combined use of multiple drugs. The comparison of the advantages and disadvantages of opioid-based and opioid-free PCIA is presented in Table 4.

**TABLE 4:** Comparison of opioid-based and opioid-free PCIA.

PCIA type	Advantages	Disadvantages
Opoid-based PCIA	1. Strong analgesic effect, significantly effective for severe pain	1. More frequent side effects
	2. Rapid onset of action	2. Side effects like nausea, constipation, respiratory depression
	3. Well-established with extensive research	3. High risk of addiction and tolerance
		4. Requires careful monitoring
Opoid-free PCIA	1. Fewer side effects (e.g., gastrointestinal reactions respiratory depression)	1. May be less effective for severe pain
	2. Lower risk of addiction	2. Potential side effects depending on the drug used
	3. Suitable for patients intolerant to opioids	3. Limited research compared to opioid-based PCIA

## 6 Disscussion

Pure opioid and opioid-free approaches represent two extremes in the application of PCIA. With the growing emphasis on multimodal analgesia, the use of pure opioid PCIA has gradually declined in clinical practice. Currently, the predominant strategy in PCIA is to minimize opioid use. This is typically achieved by combining opioids with one or two non-opioid analgesics, such as NSAIDs.

In contrast, opioid-free PCIA has emerged as a significant concept in recent years, grounded in the same principle of multimodal analgesia. This approach involves the combined use of two or more non-opioid analgesics. Recent studies have shown that for certain surgical procedures associated with mild to moderate pain, opioid-free PCIA can effectively fulfill clinical analgesic requirements. However, for surgeries that result in moderate to severe pain, continuous epidural analgesia or continuous nerve blocks are often preferred over PCIA. In cases where continuous epidural or nerve block analgesia is not suitable, opioid-free PCIA can still be effectively utilized alongside single nerve blocks or incision infiltration techniques to meet analgesic needs.

The opioid-free PCIA approach also addresses significant societal concerns, particularly in the context of the opioid abuse crisis observed in countries such as the United States (Volkow and Blanco, 2021). By ensuring that clinical pain management needs are met, the adoption of opioid-free PCIA carries considerable social significance. Moreover, for outpatient procedures or rapid recovery surgeries, opioid-free PCIA offers substantial clinical advantages (Gilbertson et al., 2021; Gosgnach et al., 2024).

## 7 Summary and prospects

Although opioids still dominate the clinical landscape of PCIA, their associated side effects are significant, especially against the backdrop of global opioid misuse. Therefore, it is imperative to advocate for and develop alternative therapies. This review, based on an extensive summary of literature, systematically outlines the clinical applications and current research status of opioid-free PCIA, including drug combination regimens, advantages, and limitations. The development of non-opioid PCIA offers a new perspective for postoperative pain management.

Currently, opioid-free PCIA is primarily used for postoperative analgesia in surgeries involving mild to moderate pain, with a predominant focus on NSAIDs combined with other non-opioid drugs. This approach not only meets analgesic needs but also significantly reduces many of the adverse effects associated with opioid analgesia, presenting a considerable advantage. However, opioid-free PCIA still has limitations for managing severe pain, and the potential side effects of opioid-free PCIA cannot be ignored. Further research and development are needed to optimize these therapies and ensure their safety and efficacy in a broader range of clinical scenarios.

Future research should focus on the development of new non-opioid drugs with stronger analgesic effects and lower risks of side effects. Additionally, exploring and optimizing combination drug strategies, especially for severe pain management, will be crucial to enhancing the therapeutic efficacy of opioid-free PCIA. More studies are needed to determine the optimal usage and dosage of various non-opioid drugs to ensure patients receive the best postoperative pain control while minimizing potential side effects. Interdisciplinary collaboration, involving experts from pharmacology, neurobiology, and clinical medicine, is essential. Conducting large-scale, multicenter clinical trials on non-opioid PCIA will further improve the effectiveness and quality of postoperative pain management. Such collaborations can better guide clinical practice and promote advancements in the field of postoperative pain management.
